# The Motor Function Evaluation of College Students’ Physical Activity State From the Perspective of Educational Psychology

**DOI:** 10.3389/fpsyg.2021.593285

**Published:** 2021-04-15

**Authors:** Sha Ge, Chao Song, Wanxiang Yao

**Affiliations:** ^1^College of Sports Science, Tianjin Normal University, Tianjin, China; ^2^Department of Kinesiology, The University of Texas at San Antonio, San Antonio, TX, United States

**Keywords:** educational psychology, static lifestyle, physical activity status, body motor function, college students, questionnaire survey

## Abstract

College students have taken part in less and less physical activities as a result of the common static lifestyle in recent years, lowering the level of motor function. This phenomenon has been a source of concern for schools and the government, and it is necessary to take corresponding measures to change it. The general motor function level of Chinese college students is explored first based on artificial intelligence and the human–computer interaction technology. The Physical Activity Questionnaire for College Students is compiled by referring to the International Physical Activity Questionnaire, and 561 students from colleges in Tianjin province are assessed based on the functional movement screen (FMS). Spearman correlation and multivariate regression analysis (MRA) are used to analyze the correlation between the motor function and physical activity status. In terms of lunch break frequency, the proportion of students having one to two lunch breaks in a week is the highest (54.15%); in terms of the frequency of doing moderate physical exercise, the proportion of students doing moderate physical exercise for 1–2 days in a week is the highest (50.61%); in terms of the frequency of doing heavy physical exercise, the proportion of students doing heavy exercise for 1–2 days in a week is the highest (47.26%); in terms of the sedentary time, the proportion of students with sedentary time more than 5 h in a day is the highest (40.61%); in terms of eye use time, the proportion of students with 8–10 h eye use time in a day is the highest (43.61%). Besides, in terms of the FMS score, the proportion of students with 13–15 FMS is the highest (48.46%). The hurdle step (21.03%), straight knee lift (22.52%), and body rotation stability (18.31%) have a relatively low proportion among the three-point items. There is a positive correlation between motor function score with the time of moderate exercise and the time of heavy exercise (*P* < 0.05). College students generally have insufficient rest time, long eye use time, and long sitting time, leading to a low level of motor function, manifested by an asymmetry between both sides of the body and poor trunk stability. It is recommended to add hurdling steps, straight knee lifts, and body rotation stability tests to college physical education courses.

## Introduction

The decline in the physical quality of college students in China has become a matter of general concern. According to the data of the National Student Physical and Health Survey, it is evident that the explosiveness, strength, and endurance of college students aged 18–22 have been slowly declining in recent years (Ghrouz et al., 2019; Penglee et al., 2019; Graupensperger et al., 2020). Scholars generally believe that it is due to the weak physical fitness awareness and the unhealthy lifestyle, that is, the static lifestyle leads to insufficient physical exercise (Kim and Cardinal, 2019; Wilson O. W. et al., 2019). Therefore, how to make students actively participate in physical exercise has become a problem faced by many colleges and universities (Goh et al., 2019). Related research shows that insufficient physical exercise is the first independent risk factor for the occurrence of chronic non-infectious diseases, and its mortality is second only to high blood pressure, smoking, and high blood sugar (Holland et al., 2019; Huberty et al., 2019). The human motor function is regulated by the close coordination of the pyramidal system, the basal nucleus, and the cerebellum, which are functionally inseparable as a whole. When the motor function is impaired, there are function disorders manifested as muscle strength and sensory and cerebellar function out of control (Acebes-Sánchez et al., 2019). Brandwayn et al. (2020) conducted a questionnaire survey on the quality of life of 18–21-year-old college students and assessed their physical fitness. The results found that regular physical exercise affected their personal health through all the following indexes: the functional reserve of the organism, the maneuverability and adaptability of the body, the lifestyle, and the resistance of the organism. Fletcher (2016) found that only 20–40% of college students engaged in a recommended amount of physical exercise. In addition, the motivation of doing physical exercise is greatly affected by friends, family, and social pressure, and they are usually motivated by external factors to do sports rather than autonomous motivation.

Human–computer interaction refers to the response of a system to a user, during which information is exchanged between humans and computers (Yıldız, 2019). Artificial intelligence is a branch of computer science involving robots, language recognition, image recognition, natural language processing, and expert systems (Fritz et al., 2020). The combination of artificial intelligence and human–computer interaction is called interactive artificial intelligence. Educational psychology is the study of human learning, the effects of educational interventions, teaching psychology, and social psychology in education (Robert et al., 2020). When it is applied to physical education, it can analyze the sports characteristics of students combining sports knowledge, sports wisdom, and sports theory, promote college students to actively participate in physical activities, and change the existing static state lifestyle, manifested by indulging in watching TV, surfing the Internet, and sitting for a long time (Klug et al., 2020; Zheng and Liu, 2020).

In the study, the Physical Activity Questionnaire for College Students based on the artificial intelligence and human–computer interaction is compiled by referring to the International Physical Activity Questionnaire, and the physical fitness of college students is assessed based on the functional movement screen (FMS). Through analysis of the current physical exercise status (number of lunch breaks in 1 week, days of moderate exercise in a week, days of heavy exercise in a week, class time in a day, sedentary time in a day, sleep time in a day, moderate exercise time in a day, heavy exercise time in a day, eye use time in a day), the current physical fitness and mental health of college students are comprehensively evaluated.

## Methodology

### Literature Analysis

College students, physical activity status, and motor function are selected as the main keywords, and the China National Knowledge Infrastructure, WANFANG, and CQVIP databases are adopted to search, and 275 relevant references are collected on college students’ health, physical activity status, artificial intelligence and human–computer interaction technology, and static lifestyle. The current foreign and domestic research progress on physical activities of college students is reviewed and summarized, so as to provide methods and theoretical support for the overall analysis of this study.

### Research Hypothesis

In the past literature analysis, most of the evaluations of college students’ motor function are deviated from the daily life of college students, which lack a joint discussion of actual life and personal physical and mental qualities. The causal relationship between college students’ physical activity status and motor function level is not clear. Owens et al. (2017) used the literature search method to collect the articles on sleep behavior of college students published from 1978 to 2016, and evaluated the factors that affected the sleep behavior of college students. It was found that insufficient sleep was related to insulin resistance, high blood pressure, diabetes, and stress, but they did not consider the differences in the individual physical qualities in different regions and different grades. Lee and Kim (2015) discussed the influence of preparations and reviews for examinations on the motor function of college students, and proved that the exam preparation and reviews effectively improved the students’ motor ability, but other factors affecting the motor function of college students were not involved. Therefore, starting from educational psychology, the relationship between physical activities and body motor function of current college students is analyzed based on artificial intelligence and human–computer interaction, to improve the theoretical quality of college students’ athletic quality and mental health. In the study, the following assumptions are taken as the preconditions.

H1: The number of lunch breaks in a week of college students significantly affects the body’s motor function.

H2: The number of moderate exercise days in a week of college students significantly positively affects the body’s motor function.

H3: The number of days of heavy activities in a week of college students significantly positively affects the motor function of the body.

H4: College students’ class time within a day significantly negatively affects the body’s motor function.

H5: College students’ sedentary time within a day significantly negatively affects the body’s motor function.

H6: The sleeping time of college students significantly affects the motor function of the body.

H7: The moderate exercise time of a day for college students significantly affects the motor function of the body.

H8: The time of heavy activities for college students significantly affects the motor function of the body.

H9: The eye use time of college students in a day significantly negatively affects the motor function of the body.

### Research Subjects

In this study, students of all grades in multiple universities in the region of Tianjin are elected as research objects. A total of 613 questionnaires are issued, and 588 are recovered. After the invalid data are excluded, there are 561 questionnaires left, and the total recovery effective rate is 91.15%.

**FIGURE 1:**
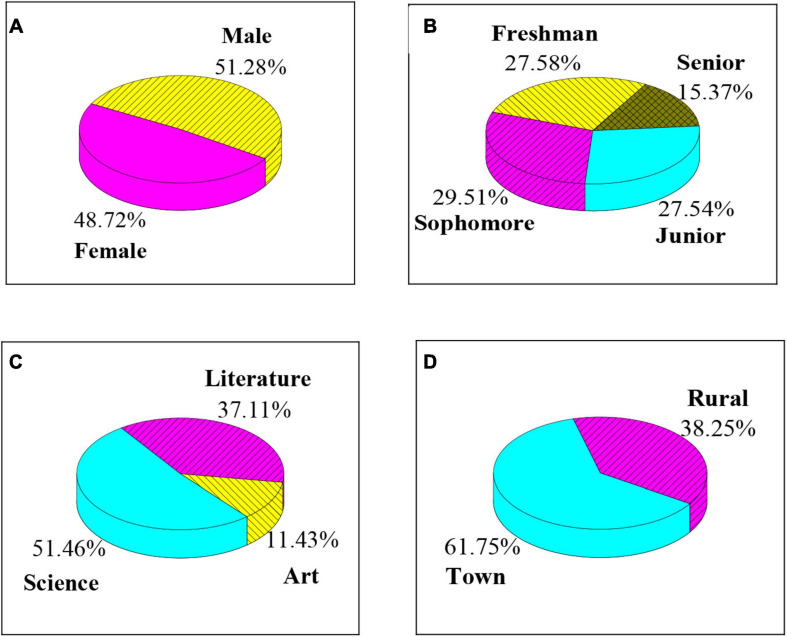
Comparison of basic information of selected college students. **(A–D)** Show the gender ratio, grade ratio, major type, and residence of college students, respectively.

Figure 1 shows the basic data of the selected college students. It revealed that the proportion of male students (51.28%) is close to the proportion of female students (48.72%); the proportion of freshmen, sophomore, junior, and senior students is 27.54, 29.51, 27.54, and 15.37%; students in science majors account for the most (51.46%), followed by students in liberal arts majors (37.11%), and arts students account for the least (11.43%); the proportion of urban students (61.75%) is significantly higher than that of rural students (38.25%).

### Questionnaire Design

Physical Activity Questionnaire for College Students: it is compiled based on the International Physical Activity Questionnaire (IPAQ) from the perspective of educational psychology (Hajar et al., 2019) and the actual life state of college students in China. The questionnaire mainly includes four parts: the personal information of the subjects, the daily rest state, the mode of entertainment activities, and the amount of physical activity. Besides, the physical activity laws, characteristics, and methods of college students were designed. The questionnaire covered a total of 32 test questions.

### Motor Function Assessment

The FMS system (Wang et al., 2017) based on the artificial intelligence and human–computer interaction, designed by American orthopedic expert Gray Cook and training expert Lee Burton in the 1990s, is adopted to test the motor function of selected college students. A four-level scoring system is used, with 0, 1, 2, and 3 points, respectively. Test 1 (over-squat squat): squat is used to detect the symmetry of the two sides of the body, the flexibility of the hips, knees, and ankles. The wooden pole above the head can detect the flexibility and symmetry of the shoulders and thoracic spine. Test 2 (hurdle step movement): hedge, knee, and ankle symmetry, flexibility, and stability can be detected through hurdle steps. Test 3 (linear lunge action): straight leg split squats can detect the flexibility of both sides of the body and the stability of the ankle and knee joints. Test 4 (shoulder flexibility movements): the shoulder flexibility test mainly reflects the abduction and rotation of the shoulder joint. Test 5 (active leg lift): the active leg lift test mainly reflects the active contraction ability of the hamstring muscle and the flexibility of the calf muscle when the pelvis was held in a fixed position. Test 6 (trunk stability push-ups): it mainly detects the stability of the trunk of the body in the sagittal plane when symmetrically using push-ups. Test 7 (body rotation stability): it mainly detects the stability and symmetry of the multi-dimensional surface of the trunk when the upper and lower limbs moved together.

### Statistical Methods

The data processing of this study is analyzed by SPSS19.0 version statistical software. The measurement data are expressed as mean ± standard deviation ($\bar{x}\pm$ s), and the count data are expressed as percentage (%). Spearman correlation and multivariate regression analysis (MRA) were used to analyze the correlation between motor function and physical activity status of college students (number of lunch breaks in a week, days of moderate exercise in a week, days of heavy activities in a week, class time in a day, sedentary time in a day, sleep time in a day, moderate exercise time of the day, heavy exercise time of the day, time of eye use within 1 day) and body mass index (BMI). *P* < 0.05 indicates that the difference was statistically significant. Origin8.0 is used to draw the figures.

## Results

### Physical Activity of College Students

**FIGURE 2:**
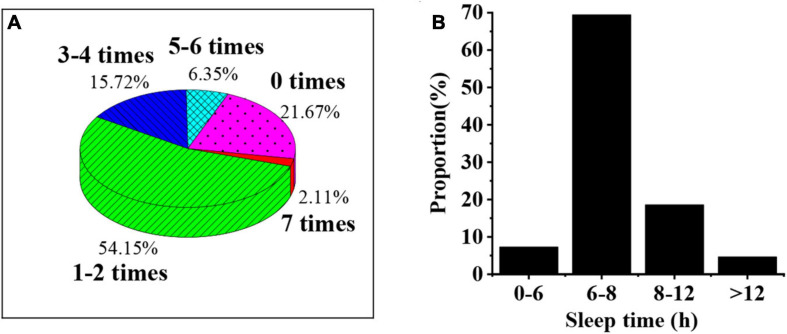
Overall rest of college students. **(A)** Was the number of lunch breaks a week for college students; **(B)** was the day’s sleep time for college students.

As shown in Figure 2, the proportion of students with one to two lunch breaks in a week is 54.15%, followed by the proportion of students with zero lunch breaks (21.67%) and the proportion of students with three to four lunch breaks (15.72%); college students with 6–8 h sleep time account for the most (69.42%), followed by those with 8–12 h sleep time (18.61%).

**FIGURE 3:**
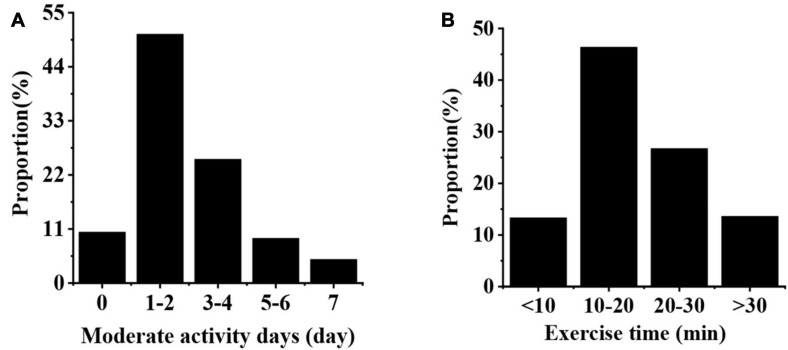
Moderate exercise status of college students. **(A,B)** Illustrate the number of moderate exercise days in a week and the moderate exercise time for college students in a day, respectively.

As shown in Figure 3, the proportion of students with 1–2 days moderate exercise in a week is the highest (50.61%), followed by those with 3–4 days (25.17%) and 7 days (4.79%) moderate exercise; students with an activity time of 10–20 min in a day account for the largest proportion (46.37%), followed by those with 20–30 min (26.72%), and the proportion of those with more than 30 min activity time is the smallest (13.60%).

### Physical Activities at the Learning Level

**FIGURE 4:**
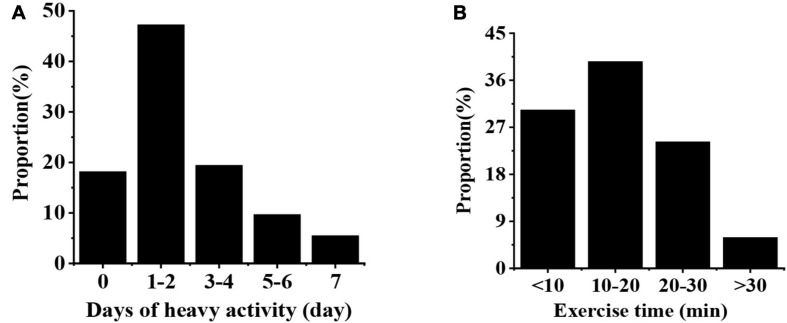
The status of heavy activities of college students. **(A)** Illustrates the number of days of heavy activities for college students, and **(B)** Indicates the day of heavy activities for college students.

As shown in Figure 4, the proportion of students with 1–2 days heavy exercise in a week is the largest (47.26%), followed by the proportion of those with 3–4 days (19.42%) and the proportion of those with 7 days of activity (5.48%); the proportion of college students who spent 10–20 min on strong activities in a day is the highest (39.58%), followed by the proportion of those who spent 0 min (30.31%), and the proportion of those who spent more than 30 min is the least (5.91%).

**FIGURE 5:**
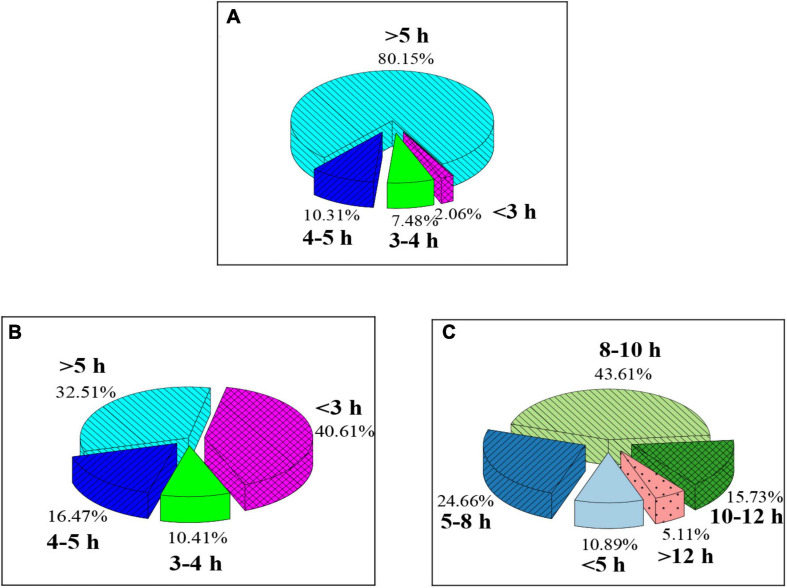
Physical activity of college students at the learning and entertainment level. **(A–C)** Indicate the class hours, sedentary time, and eye use time of college students in one day, respectively.

As shown in Figure 5, college students with class hours of more than 5 h have the highest proportion (80.15%), and the proportion of those with class hours of less than 3 h is very small (2.06%); college students with more than 5 h of sedentary time have the highest proportion (40.61%), followed by the proportion of those with 4–5 h sedentary time (32.51%), and the proportion of those with class hours of less than 3 h is very small (10.41%); the proportion of college students with 8–10 h of eye use time in a day is the highest (43.61%), followed by the proportion of those with 5–8 h (24.66%) and 10–12 h (15.73%) of eye use time.

### Motor Function Evaluation Results

**FIGURE 6:**
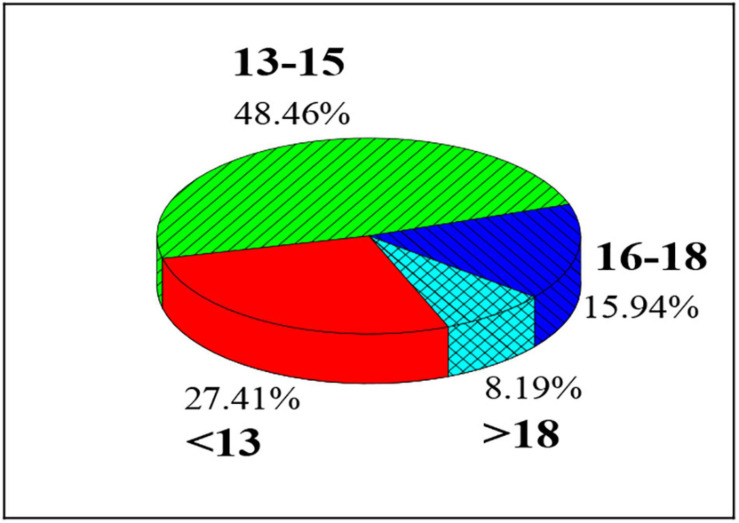
Proportion of college students’ FMS segments.

As shown in Figure 6, the proportion of students with total FMS scores of 13–15 points is the highest (48.46%), followed by the proportion of those with less than 13 points (27.41%) and those with 16–18 points (15.94%), and the proportion of those with more than 18 points is the least (8.19%).

**FIGURE 7:**
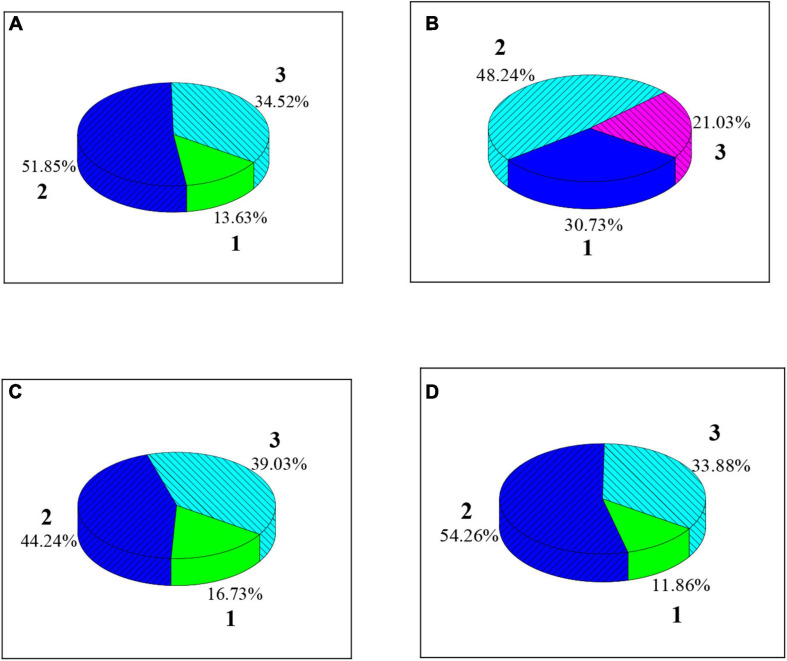
The proportion of different scores of the four test items of FMS for college students. **(A)** Was a squat over the top; **(B)** Was a hurdle; **(C)** Was a straight lunge; **(D)** Was shoulder flexibility.

**FIGURE 8:**
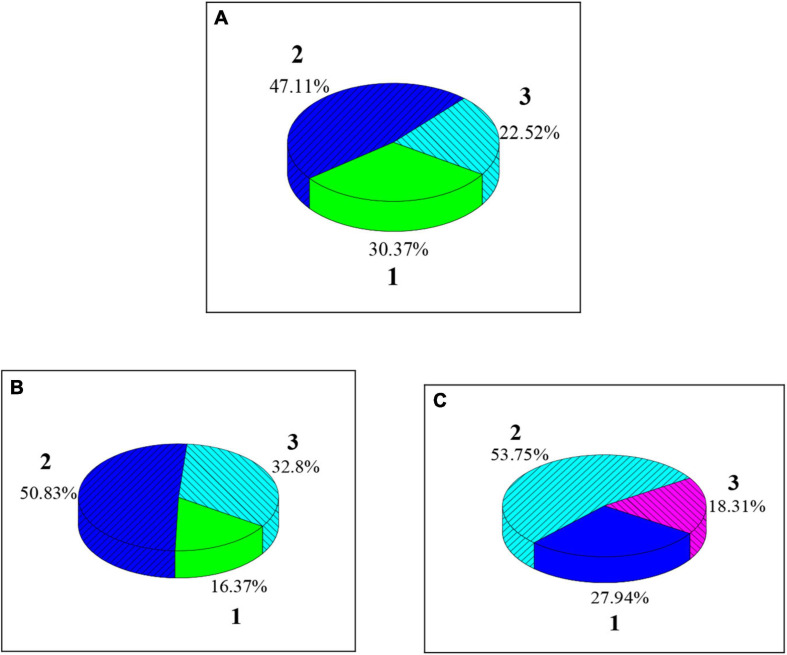
The proportion of different scores of the three test items of college students’ FMS. **(A–C)** Disclose the scores of straight knee lift, torso flexibility, and body rotation flexibility, respectively.

As shown in Figures 7, 8, among the college students’ FMS test items, test items scoring two points have the largest proportion among the seven. High squats (34.52%), straight lunges (39.03%), shoulder flexibility (33.88%), and torso flexibility (32.80%) have relatively high proportions among the three-point items; hurdles (21.03%), straight knee lift (22.52%), and body rotation stability (18.31%) have relatively low proportions among the three-point items.

### BMI of Selected College Students

**FIGURE 9:**
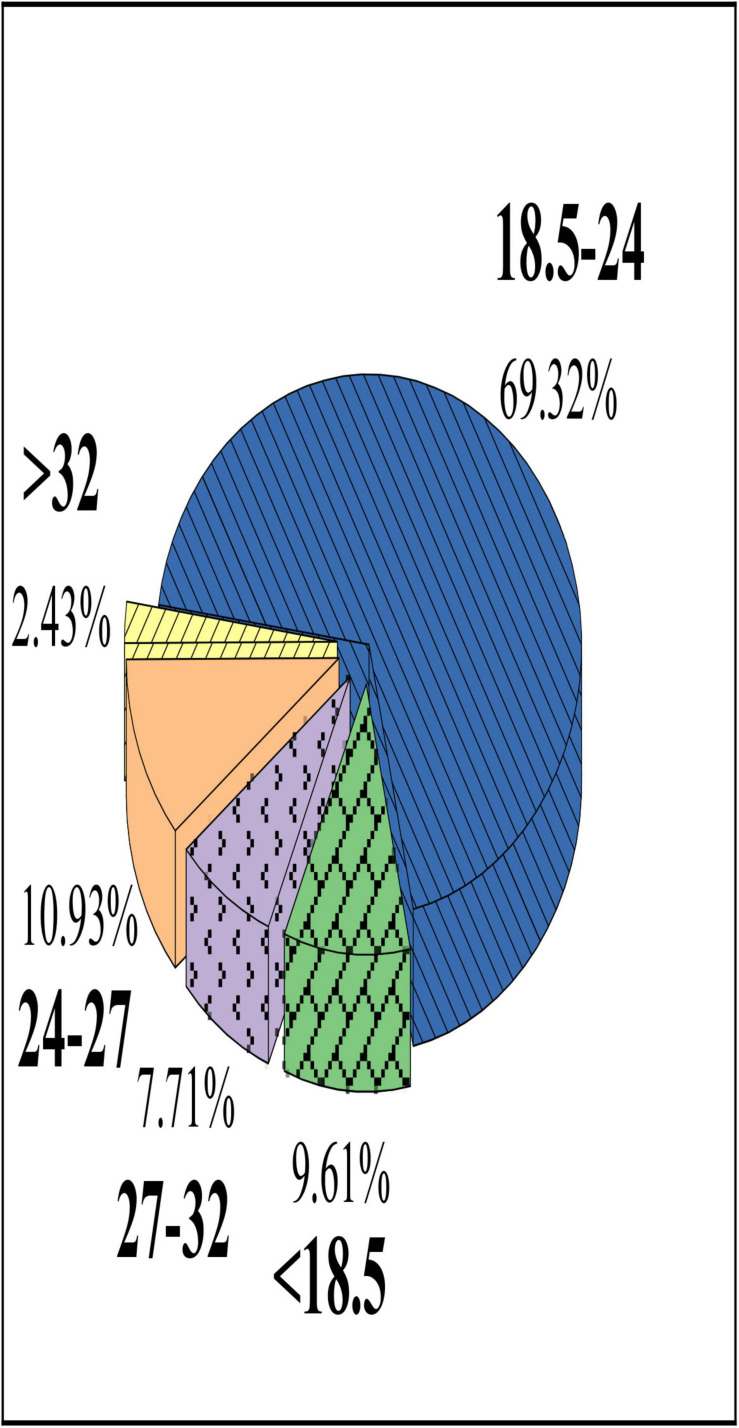
BMI analysis of college students.

As shown in Figure 9 below, the proportion of college students with a BMI of 18.5–24 kg/m^2^ is 69.32%, the proportion of those with more than 24 kg/m^2^ is 21.07%, and the proportion those with less than 18.5 kg/m^2^ is 9.61%.

### Motor Function Scores in Demographic Variables

Table 1 suggests that there is no significant difference in college students’ FMS scores in terms of gender and place of residence (*P* > 0.05). FMS scores of college students have a significant difference in grades, and the FMS scores of junior students are significantly higher than those of students in other grades (*P* < 0.05). FMS scores of college students are significantly different in terms of majors, and the FMS scores of art students are significantly higher than those of students in other majors (*P* < 0.05).

**TABLE 1:** Basic situation of selected college students.

**Variable**	**Classification**	**FMS score**	**χ ^2^**	***P*-value**
Gender	Male	14.17 ± 5.82	2.175	0.079
	Female	13.72 ± 6.02		
Grade	Freshman	15.11 ± 5.87	5.873	0.018
	Sophomore	13.26 ± 6.21		
	Junior	15.33 ± 4.98		
	Senior	13.42 ± 5.47		
Major	Science	13.23 ± 6.11	7.328	0.007
	Liberal arts	13.42 ± 6.72		
	Art	15.59 ± 5.47		
Place of residence	Town	14.48 ± 5.71	1.285	0.063
	Rural	13.91 ± 5.93		

### Correlation Between BMI and Motor Function Score

As shown in Table 2, Spearman correlation analysis shows that there is a significant negative correlation between college students’ motor function score and BMI (*r* = -5.382, *P* < 0.05). MRA is conducted by taking BMI as an independent variable and motor function score as the dependent variable. As shown in Table 3, the regression coefficient of BMI and motor function score of college students was −0.488, which has a significant negative effect (*P* < 0.05).

**TABLE 2:** Spearman correlation analysis between BMI and score of motor function.

**Variable**	**Motor function score**	
	***r***	***P*-value**
BMI	−5.382	0.008

**TABLE 3:** Multivariate regression analysis of college students’ BMI and sports behavior.

**Variable**	**Motor function score**		
	**Regression coefficients**	***t*-value**	***P*-value**
BMI	−0.488	5.724	0.022

### Correlation of Physical Activity Level and Motor Function Score

Table 4 indicates that there is no significant correlation between college students’ motor function scores and the number of lunch breaks in a week, the number of moderate exercise days in a week, the number of heavy exercise days in a week, the class time in a day, and the sedentary time in a day (*P* > 0.05); college students’ motor function scores are significantly correlated with sleep time in a day, moderate exercise time in a day, heavy exercise time in a day, and eye use time in a day (*P* < 0.05).

**TABLE 4:** Spearman correlation analysis of degree of physical activity and motor function score of college students.

**Variable**	**Motor function score**	
	***r***	***P*-value**
Number of lunch breaks in a week	2.444	0.053
Sleep time in a day	6.041	0.018
Days of moderate exercise in a week	1.769	0.051
Days of heavy exercise in a week	2.641	0.062
Moderate exercise time of the day	4.227	0.004
Heavy exercise time of the day	3.741	0.021
Class time in 1 day	1.711	0.064
Sedentary time in 1 day	2.640	0.059
Eye time in 1 day	−5.275	0.013

As shown in Table 5, the regression coefficient of college students’ motor function score and sleep time within a day is 0.258, and there is no significant impact (*P* > 0.05); the regression coefficients of college students’ motor function score and 1-day moderate exercise time and heavy exercise time of one day are 0.531 and 0.527, respectively, and there are significant positive effects (*P* < 0.05); the regression coefficient of college students’ motor function score and eye use time within a day is −0.489, and there is a significant negative effect (*P* < 0.05).

**TABLE 5:** Multivariate regression analysis of the degree of physical activity and motor function score of college students.

**Variable**	**Motor function score**		
	**Regression coefficients**	***t*-value**	***P*-value**
Sleep time in a day	0.258	2.642	0.056
Moderate exercise time of the day	0.531	5.118	0.014
Heavy exercise time of the day	0.527	4.862	0.028
Eye time in 1 day	−0.489	5.217	0.042

## Discussion

The development of students’ physical and psychological qualities during university is an important factor determining the direction of future life. The general static lifestyle has led to the decline of college students’ physical function, and it is a hot topic to use artificial intelligence and human–computer interaction technology to obtain body-related information (Zhang et al., 2019). Therefore, the Physical Activity Questionnaire for College Students is compiled by referring to the International Physical Activity Questionnaire. Students’ physical fitness is assessed based on FMS. It is found that college students with one to two lunch breaks in a week account for the largest proportion (54.15%), followed by the proportion of those with zero lunch breaks (21.67%) and the proportion of those with three to four lunch breaks (15.72%), which is similar to the survey results of Haidar et al. (2019). Rest is the basic condition for improving the body’s health index and completing daily activities. Good sleep can ensure the body’s athletic quality and mental health. However, the results show that many college students have a bad habit of not taking a lunch break during the day, which cannot reach the normal rest level. College students with 6–8 h sleep time in a day account for the most (69.42%), followed by 8–12 h (18.61%). From a psychological point of view, college students do not attach importance to the development of a sleep habit of 8 h a day and incline to sleep until they feel sleepy, which is different from the research results of Wilson O. W. A. et al. (2019).

Abundant amount of daily activities is conducive to improving the motor function of college students. It is found in the study that college students with 1–2 days moderate exercise have the largest proportion (50.61%); the proportion of students with activity time between 10 and 20 min is the highest (46.37%), which is different from the results of Liu et al. (2019). The reason may be that moderate activities generally refer to sports such as badminton, table tennis, and cycling, indicating that the college student group lacks additional moderate activities in addition to some daily activities. Therefore, it is necessary to strengthen the overall amount of moderate physical activity of college students (Win et al., 2019). College students with 1–2 days of strong activities account for the most (47.26%); the proportion of students with strong activities in 10–20 min is 39.58%, which is similar to the research results of Chan et al. (2019), indicating that the college student lack normal heavy exercise. As a result, the proportion of college students with moderate to strong physical activity is low. It is a problem that schools need to pay attention to. The proportion of college students with more than 5 h of class in a day is the highest (80.15%), and the proportion of those with less than 3 h is very small (2.06%). Because of the constraints of our education system, college students have to spend more time and energy completing schoolwork, which is also one of the factors that reduce physical activity of college students (Barkley et al., 2019). The students with eye use time of 8–10 h in a day have the highest proportion (43.61%), which is the same as the results of Ouyang et al. (2020). As entertainment activities such as computers, mobile phones, tablets, and games take up a lot of time for students, the eye use time is also prolonged greatly. Therefore, colleges should consider formulating strict physical activity programs to improve the psychological quality and athletic literacy of college students (Corder et al., 2019). The BMI can reflect the individual’s health status and the degree of fatness and thinness. It is found that the proportion of college students with a BMI of 18.5–24 is 69.32%, and the proportion of those with more than 24 is 21.07%, indicating that most of the students’ body weight was normal, but there are still more than 20% with obesity. Therefore, college students need to pay more attention to physical health and avoid obesity.

The proportion of students with a total FMS of 13–15 points is the highest (48.46%), followed by the proportion of those with less than 13 points (27.41%) and those with 16–18 points (15.94%), which is similar to the results of Lang et al. (2019). It indicates that most students’ overall motor function level is barely qualified, and a considerable number of people still suffer from low motor function. From the specific test items, squats (34.52%), straight lunges (39.03%), shoulder flexibility (33.88%), and torso flexibility (32.80%) account for a relatively high proportion among three-point items; hurdle steps (21.03%), straight knee lifts (22.52%), and body rotation stability (18.31%) account for a relatively low proportion among three-point items, which show that the overall ability of college students is better to complete over-the-top squats, straight lunge, and shoulder flexibility, but is poor to complete the hurdle step, straight knee lift, and body rotation stability test. The reason may be that the spine distortion caused by the students’ incorrect sitting posture for a long time affects their mental and physical health. The hurdle step, straight knee lift, and body rotation stability test can be added to the college physical education test to improve the student’s sports psychology quality (Chen, 2019; Saller and Khaled, 2019).

FMS scores of college students have significant differences in grades, and the FMS scores of junior students are significantly higher than those of other grades (*P* < 0.05), which is different from the results of Gong et al. (2019). It may be due to the academic pressure of freshman and sophomore students. The lack of sufficient after-school time results in poor average motor function, while the senior students face some pressures such as graduation, postgraduate examination, and employment, which lead to the reduction of daily physical activity, thus affecting the body’s motor function (Zeng et al., 2017). FMS scores of college students are significantly different in terms of majors, and the FMS scores of art students are significantly higher than those of students in other majors (*P* < 0.05), which is because art students have rich daily physical activities and more spare time, thus maintaining a high level of athletic ability (Suraya et al., 2017; Feng and Chen, 2020). MRA discloses that the regression coefficient of college students’ BMI and motor function score is −0.488, which has a significant negative effect (*P* < 0.05), indicating that the college student’s BMI is negatively correlated with their motor function level. Regression coefficients of college students’ motor function scores and moderate exercise time in a day and heavy exercise time in a day are 0.531 and 0.527, respectively, and there are significant positive effects (*P* < 0.05), showing that the activity of college students is positively correlated with the improvement of their motor function within a certain range. Thus, H7 and H8 are proved to be true. The regression coefficient of the motor function score of college students and the eye use time within a day is −0.489, which has a significant negative effect (*P* < 0.05), suggesting that the use frequency of computers and mobile phones can be shortened by reducing the eye use time, which is conducive to improving students’ psychological quality of sports. Thus, H9 is proved to be true (Fuertes and Hoffman, 2016). In addition, college students’ motor function scores are not significantly correlated to the number of lunch breaks in a week, the number of days of moderate exercise in a week, the number of days of heavy exercise in a week, the class hours in a day, the sedentary time in a day, and the sleep time in a day (*P* > 0.05), which indicates that the hypotheses H1, H2, H3, H4, H5, and H6 are not true.

## Conclusion

The College Students Physical Activity Questionnaire is compiled by referring to the International Physical Activity Questionnaire, and the physical fitness of college students is assessed based on FMS. Sedentary habit is one of the basic factors leading to students’ low-level motor function, manifested by asymmetry of both sides of the body and poor trunk stability. It is recommended to add hurdles, straight knees, and body rotation stability tests to college physical education courses. However, the samples are selected from the same area, which may reduce the power of the study. An expanded sample size involving multiple areas in the future is necessary to strengthen the findings of the study. In conclusion, the study provides a theoretical basis for the improvement of college students’ mental health.

## Data Availability Statement

The raw data supporting the conclusions of this article will be made available by the authors, without undue reservation.

## Ethics Statement

The studies involving human participants were reviewed and approved by the Tianjin Normal University Ethics Committee. The patients/participants provided their written informed consent to participate in this study. Written informed consent was obtained from the individual(s) for the publication of any potentially identifiable images or data included in this article.

## Author Contributions

All authors listed have made a substantial, direct and intellectual contribution to the work, and approved it for publication.

## Conflict of Interest

The authors declare that the research was conducted in the absence of any commercial or financial relationships that could be construed as a potential conflict of interest.
